# Lateralized hypostasis of the head on post mortem CT (PMCT) scanning of decomposed bodies—a marker for terminal position

**DOI:** 10.1007/s12024-023-00698-z

**Published:** 2023-08-25

**Authors:** Roger W. Byard

**Affiliations:** 1 Forensic Science SA, 21 Divett Pl, Adelaide, SA 5000 Australia; 2 School of Biomedicine, University of Adelaide, Frome Rd, Adelaide, South Australia 5005 Australia

**Keywords:** Decomposition, Putrefaction, Hypostasis, PMCT, Position of body

## Abstract

Post-mortem CT (PMCT) scanning was performed on the bodies of two decomposed adult males who had died of natural causes. The bodies both showed changes of marked decomposition with a prominent swelling of tissues on one side of the head compared to the other. A review of police statements confirmed that this matched the positions of the bodies when they were found. Thus, post-mortem hypostasis of putrefactive fluids correlated in both cases with the positions that the bodies had been in when first located. This may be a simple way of identifying or confirming the positions of decomposed bodies after death. It may also assist in indicating whether a body has been moved after putrefactive fluid hypostasis has developed.

## Case studies

To eliminate identifiable case details, only descriptions of post-mortem intervals, body positions, and CT findings of the heads have been included in the following text.

### Case 1

**Fig. 1:**
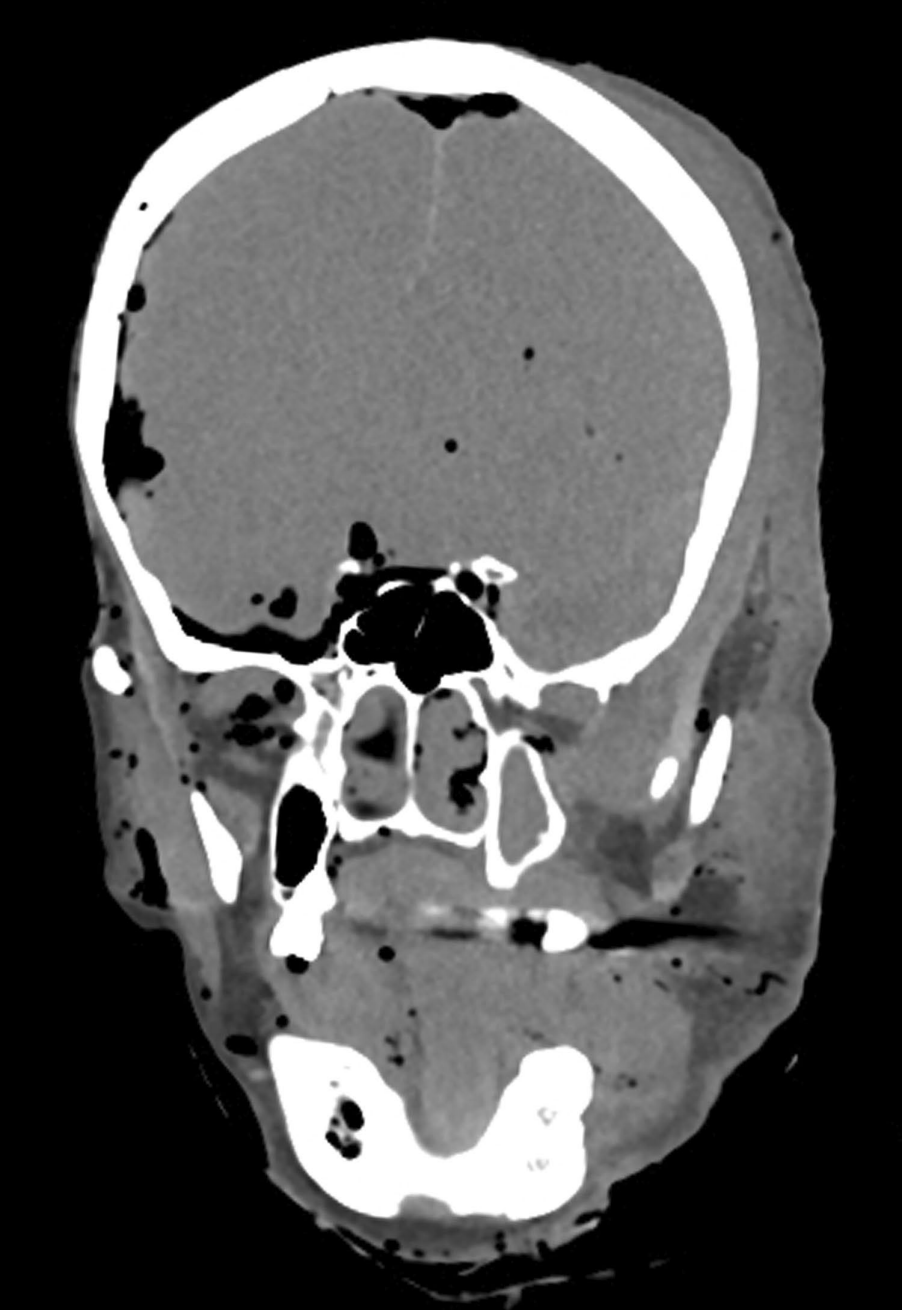
Coronal section of the head in a decomposed adult body revealing marked fluid accumulation on the left side of the head compared to the right. This finding correlated with the police report documenting the position of the body on its left side at the scene

An adult male who died of natural causes had not been seen for approximately 3 weeks. He was found on the floor of his house. The body showed changes of marked decomposition with insect infestation. PMCT scanning also demonstrated changes of marked decomposition with prominent swelling of tissues on the left side of the head (Fig. 1). A review of the police statement showed that the body had been found lying on the left side. There were no injuries detected on scanning or at the autopsy.

### Case 2

**Fig. 2:**
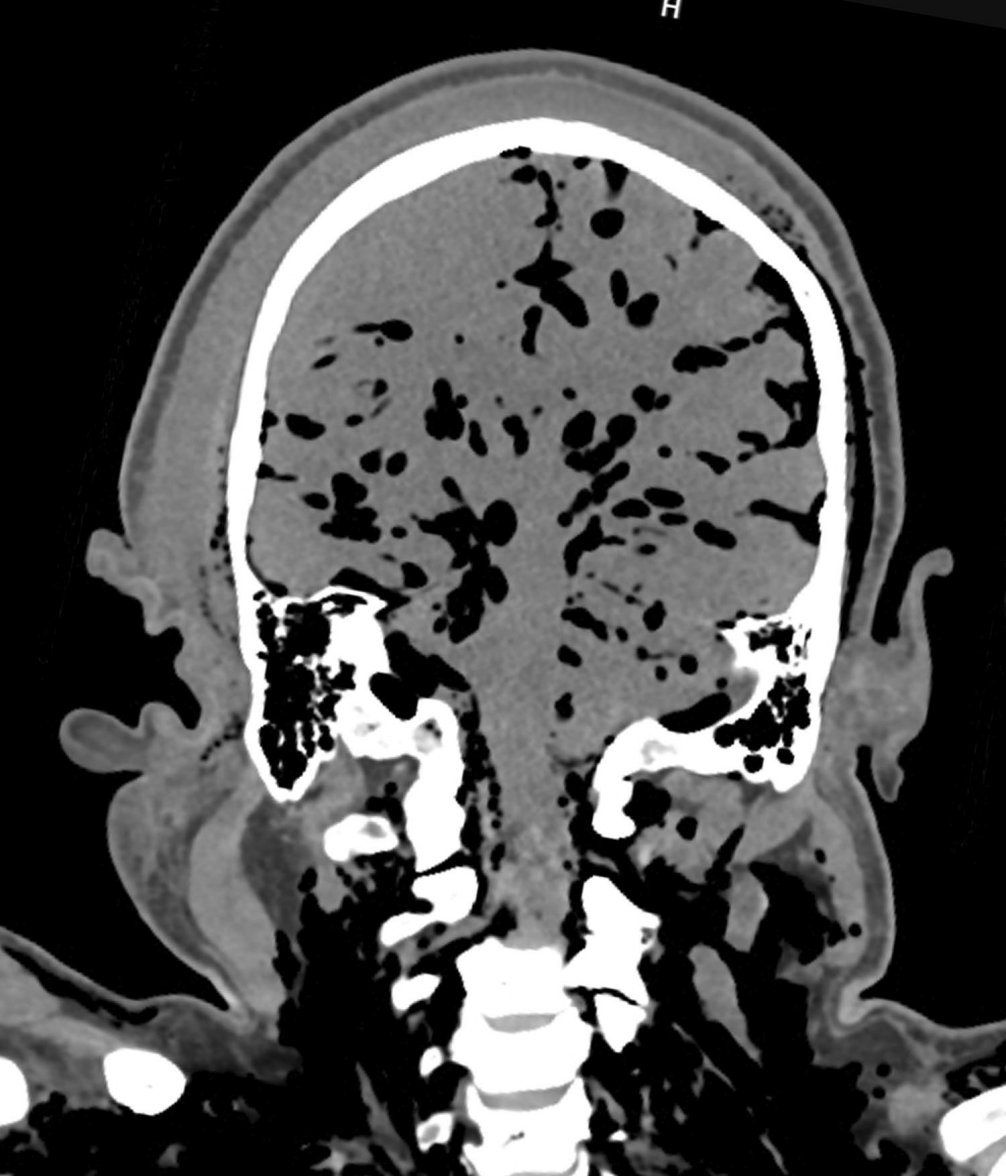
Coronal section of the head in a decomposed adult body revealing marked fluid accumulation on the right side of the head compared to the left. This finding correlated with the police report documenting the position of the body on its right side at the scene

A second adult male who also died of natural causes had not been seen for approximately 11 days. He was found on a bed in a caravan. The body showed changes of established decomposition. PMCT scanning again demonstrated changes of decomposition with prominent swelling of tissues on the right side of the head (Fig. 2). A review of the police statement showed that the body had been found lying on the right side. There were no injuries detected upon scanning or at the autopsy. The bodies had been stored on their backs in the morgue’s refrigerated area for between 3 and 7 days, respectively, before autopsy.

## Discussion

Hypostasis generally refers to the accumulation of fluid or blood in the lower parts of the body or organs under the influence of gravity. The most commonly encountered form of hypostasis at autopsy is lividity, where blood pools within the dermal capillaries in dependent tissues. This was first described by Ploucquet in 1787 [1]. As with many post-mortem changes, there is great individual variability in the time course for its development, with lividity generally appearing within 30 min to 3 h after death. After some time (~ 18–24 h), it becomes fixed, meaning that it will not change if the position of the body is altered, and it will not blanch under pressure [1, 2].

If a body is resting against a particular surface or object, or is compressed by clothing, so-called patterned lividity may be found, which records the nature of the contact surface/material [3]. Although, in the past, predictions regarding the time of death have been made by assessing lividity, the most useful features of lividity are to demonstrate that a body has, or has not, been moved after death, or to indicate the nature of the surface that the body was resting on.

Problems may occur if lividity is confused with bruising, leading, for example, to the misinterpretation of normal post-mortem hymenal lividity as a sign of sexual trauma [4]. The differentiation of possible bruising from lividity can usually be achieved by incising the areas, as lividity is characteristically an intravascular phenomenon [5]. However, it must be recognized that situations may arise where post-mortem extravascular hemorrhages may be caused by an increase in hydrostatic pressure in autolyzing venous plexuses under the influence of gravity. This may result in artefactual hemorrhagic lividity in the soft tissues of the neck potentially mimicking antemortem injury [6] and in the posterior pharyngeal wall [7].

Lividity is not usually a feature of marked decomposition, as the skin color will have altered to deep red/purple or green, with skin blistering and sloughing. Insect activity may also have removed much of the skin surface [8]. This does not mean, however, that hypostasis will not be encountered in putrefactive and autolytic bodies as decomposition results in the liquefaction of organs and soft tissues with the pooling of this fluid under gravitational influence [9].

The assessment of hypostasis by PMCT has been previously undertaken, demonstrating it as a common phenomenon intracranially. Again, however, there is a risk of misinterpretation with the possibility of the pooling of blood being incorrectly diagnosed as subarachnoid hemorrhage [10]. Post-mortem imaging has also been used to evaluate intracardiac and neonatal hypostasis as a potential measure of post-mortem interval [11, 12].

In the current report, the examination of coronal sections of the head in two decomposed bodies revealed marked fluid accumulation on one side of the head/scalp compared to the other (Figs. 1 and 2). This suggested post-mortem hypostasis of putrefactive fluids and was correlated in both cases with the positions that the bodies had been found in when first located. Thus, this relatively simple additional observation may be a useful way of identifying or confirming the position that a decomposed body has been in for some time after death. It may also potentially be of use if a body has been moved after such fluid accumulation has occurred.
